# Vitamin D, Adiponectin, Oxidative Stress, Lipid Profile, and Nutrient Intakes in the Females with Acne Vulgaris: A Case-Control Study

**DOI:** 10.31661/gmj.v8i0.1515

**Published:** 2019-08-09

**Authors:** Mahsa Moazen, Zohreh Mazloom, Farideh Jowkar, Nasrin Nasimi, Zahra Moein

**Affiliations:** ^1^Student Research Committee, School of Nutrition and Food Sciences, Shiraz University of Medical Sciences, Shiraz, Iran; ^2^Nutrition Research Center, School of Nutrition and Food Sciences, Shiraz University of Medical Sciences, Shiraz, Iran; ^3^Molecular Dermatology Research Center, Department of Dermatology, Shiraz University of Medical Sciences, Shiraz, Iran

**Keywords:** Acne Vulgaris, Anti-Inflammatory Agents, Oxidative Stress, Lipids, Nutrition Assessment

## Abstract

**Background:**

Acne vulgaris is a dermatological disorder that is related to inflammation and oxidative stress. Recent studies have also suggested diet as a potential reason for acne. Considering the inconsistency of the few previous reports, the present study aimed to determine the levels of vitamin D, adiponectin, oxidative stress, lipid profile and nutrient intakes in females with acne.

**Materials and Methods:**

Forty females with acne vulgaris and 40 age-matched healthy females were included in this study in Shiraz, Iran. Data on their sociodemographic status, acne history, and anthropometric indices were collected. Blood samples were taken to determine the levels of 25-hydroxyvitamin D, adiponectin, malondialdehyde, total antioxidant capacity, and lipid profile. Three 24-hour dietary recalls were also obtained from each of the individuals to evaluate nutrient intakes. Data were analyzed using the Chi-square test, Independent-samples t-test or Mann–Whitney U-test, and Logistic Regression.

**Results:**

Patients with acne had a significantly higher family history of acne compared to controls (P=0.006). Serum level of malondialdehyde was significantly higher in cases (P=0.01), while high-density lipoprotein cholesterol was significantly lower (P=0.02). Moreover, significantly lower fiber intake was observed in cases compared with controls (P=0.007). In the multivariate analysis, a family history of acne and increased malondialdehyde levels were risk factors for acne, whereas a higher fiber intake was protective.

**Conclusion:**

Family history of acne, oxidative stress, dyslipidemia, and lower dietary fiber intakes may play a role in acne pathogenesis. An early assessment of these parameters may be useful for planning treatment procedures.

## Introduction

Acne vulgaris is a common dermatological disorder of the pilosebaceous unit. It has multifactorial pathogenesis that results in immune-mediated inflammation [1, 2]. Acne can elicit psychological disturbances such as depression or anger and may decrease the quality of life [3]. Vitamin D apart from its well-known significance in calcium homeostasis can influence the innate and adaptive immune systems and has anti-inflammatory properties [4, 5]. Adiponectin is another anti-inflammatory molecule that is mainly released from the adipose tissue. It suppresses proinflammatory cytokines, increases insulin sensitivity, and also has antioxidant effects [6, 7]. However, results from the few studies that have been carried out on serum levels of vitamin D [5, 8] and adiponectin [6, 9, 10] in acne patients are controversial. Furthermore, it has been reported that oxidation of sebum and oxidative stress in the pilosebaceous unit provide an environment that may lead to some pathogenic factors which result in acne [11]. Therefore, in recent studies, oxidative stress and antioxidant status have been investigated in patients with acne, and free radicals have been suggested in the pathophysiology of the disease [12-14]. Besides, androgens, which have an important role in the development of acne, are generated by serum cholesterol. Androgens also raise hepatic lipase activity. If the activity of this enzyme is sufficiently high, it may ultimately increase low-density lipoprotein cholesterol (LDL-C) levels [15]. Some studies have indicated a deterioration in the lipid profile of acne patients, but the alterations in the specific components of serum lipids are not identical [15, 16]. Recent studies have described diet as a potential reason for acne. A relationship between acne and diet used to be rejected for years, partially based on the findings of the early investigations. However, the original study was re-examined, and methodological faults were found with it. Subsequent studies demonstrated the role of dairy products and high glycemic load diets in the etiology of acne [17]. Nevertheless, very few studies have explored individual nutrient intakes in these patients, and the results have been inconsistent [18, 19]. Considering the insufficiency and discrepancy of the findings of the previous investigations, the aim of the current study was to determine the levels of vitamin D, adiponectin, markers of oxidative stress and lipid profile as well as the amount of nutrient intakes in females with acne vulgaris and comparing them with controls, to elucidate the mechanisms of acne pathogenesis.

## Materials and Methods

### Sample Size Calculation

The minimum sample size was calculated based on the desired type I error of 0.05 and a power of 95% according to a previous study [12]. A total of 80 participants were considered satisfactory for the current research.

### Patients

The present research was designed as a case-control study and was carried out in the city of Shiraz, Iran, in 2017. Forty female patients with acne vulgaris were recruited from Motahari and Emam Reza clinics by convenience sampling method. A dermatologist confirmed the presence of acne vulgaris and the severity of the disease was determined according to the Global Acne Grading System [20]. Forty healthy females were also enrolled from the same clinics where the cases arose and were matched to cases by age (+/-2 years). Considering that seasonal changes can affect vitamin D levels, for each recruited case, the matched control was selected in the pertinent season.

### Inclusion and Exclusion Criteria

Inclusion criteria were those females in the age range of 15-35 years and diagnosed with moderate to severe acne vulgaris. Exclusion criteria included the presence of a systemic disease, taking vitamin-mineral supplements, following unusual diets, tobacco use, pregnancy, and lactation. Patients who had taken any medications to treat acne for at least three months prior to entry into the study were also excluded [12].

### Data Collection

All the participants were asked to fill out a questionnaire concerning their sociodemographic and acne history information. Afterward, they were weighed on a standard scale (Seca, Germany) to the nearest 0.1 kg, while they were in light clothing and had no shoes on. Their heights were also measured by a stadiometer to the nearest 0.1 cm. Their body mass indexes (BMI) were then calculated. Subsequently, venous blood samples of the individuals were drawn after 12 hours of fasting and were centrifuged. Collected serums were analyzed for 25-hydroxyvitamin D, adiponectin, markers of oxidative stress and lipid profile. Enzyme-linked immunosorbent assay (ELISA) was used to assess 25-hydroxyvitamin D and adiponectin levels using commercially available kits (Monobind Inc, USA, and IBL, Germany, respectively). The concentrations of malondialdehyde (MDA) were measured spectrophotometrically by thiobarbituric acid-reactive substances (TBARS) method. Total antioxidant capacity (TAC) levels were determined by an enzymatic, colorimetric method by an available kit (ZellBio, Germany). The lipid profile including triglycerides, total cholesterol, high-density lipoprotein cholesterol (HDL-C), and LDL-C were also measured by enzymatic colorimetric methods (Pars Azmoon, Iran) on an autoanalyzer (BT-1500, Italia). In order to determine the prevalence of vitamin D deficiency in each group, the cut-off point of 20 ng/ml for 25-hydroxyvitamin D [21] was applied. Moreover, as a valid method [22], three 24-hour dietary recalls were obtained from each participant by trained nutritionists. These included two weekdays and one weekend day. The first recalls were collected by in-person interviews, and the two others were telephone-administered. Their daily energy and nutrient intakes, with a focus on macronutrients and antioxidant micronutrients, were then determined using Nutritionist IV software (First Data Bank, San Bruno, CA; version 3.5.2).

### Ethical Statements

The study protocol was approved by the Ethics Committee of Shiraz University of Medical Sciences (Ethics code: IR.SUMS.REC.1394.S873) and was in accordance with the Helsinki Declaration. All the individuals received a detailed description of the study and signed an informed consent to participate.

### Statistical Analysis

Data in each group were expressed as mean ± standard deviation (SD) for quantitative variables, and frequency and percentage for qualitative ones. The Chi-square test was used for analyzing the qualitative data. Normality of the numerical variables was assessed by the Kolmogorov–Smirnov test. Differences between the groups were determined by the Independent-samples t-test or the Mann–Whitney U-test, depending on the distribution of the data. These analyses were performed with the Statistical Package for the Social Sciences (SPSS) software (SPSS Inc., Chicago, IL, USA), version 15. Univariate logistic regression models were also used to predict factors affecting acne. Subsequently, those variables with a significance level of <0.2 were entered into multivariate logistic regression models to eliminate potential confounders. Logistic regression was done by Stata software, version 13.0. P-values of <0.05 were considered statistically significant.

## Results

Forty individuals participated in each of the groups. Among the patients, 31 (77.5%) were diagnosed with moderate acne, and only nine (22.5%) had the severe form. Sociodemographic data, acne history information and anthropometric indices of the participants are presented in Table-1. The age ranges of the cases and controls were 16 to 32 and 18 to 33 years, respectively. According to the Chi-square test, Independent-samples t-test or Mann–Whitney U-test, there were no significant differences between acne and control groups in terms of age, marital status, educational level, and income status as well as weight, height, and BMI. However, the patients had a significantly higher family history of acne in first-degree relatives than did healthy individuals (P=0.006). Results on the biochemical markers showed that serum MDA levels were significantly higher (P=0.01) and HDL-C levels were significantly lower (P=0.02) in acne patients compared to healthy controls. Nonetheless, no considerable differences were found between the groups with regard to 25-hydroxyvitamin D, adiponectin, TAC, triglycerides, total cholesterol, and LDL-C levels. The prevalence of vitamin D deficiency was also similar between the groups (Table-2). The findings concerning daily nutrient intakes demonstrated a significant lower fiber consumption in cases than in controls (P=0.007). However, the two groups were similar in terms of their energy and macronutrient intakes (i.e., total carbohydrate, protein, and fat) as well as the intake of micronutrients including vitamin A, beta- carotene, vitamin E, vitamin C, selenium, zinc and vitamin D ( Table-3). After adjustment for confounders by logistic regression, presence of family history of acne and higher MDA levels were associated with increased odds of developing acne (odds ratio [OR]=4.00, P=0.03 and OR=2.15, P=0.047, respectively), while higher fiber intake was shown to be a protective factor (OR=0.87, P=0.04, Table-4).

## Discussion

The results of the present study demonstrated that there were no significant differences between acne and control groups in terms of 25-hydroxyvitamin D levels or the prevalence of vitamin D deficiency. This finding is in accordance with a recent study that had determined the level of this vitamin in acne patients [8]. However, another research had reported a remarkably higher prevalence of vitamin D deficiency in acne patients than in healthy individuals [5]. It has been indicated that human cultured sebocytes express vitamin D receptors and are involved in the metabolism of active vitamin D analogs. Vitamin D analogs change the activity of sebaceous glands and as a result, may serve as promising compounds for curing acne [23]. High prevalence of vitamin D deficiency has been reported in the Iranian population [24]. Therefore, the existence of low levels of this vitamin in both healthy people and those with acne may be a reason for observing no significant differences between the two groups in this study. The high prevalence of 62.5% for vitamin D deficiency in the current study supports this opinion. In terms of adiponectin levels, the findings of this study did not show any significant differences between acne and control groups. In this context, results from the previous investigations are thoroughly controversial. For instance, in the study by Ozuguz *et al*. [9] adiponectin concentrations did not differ between non-obese acne patients and controls. Nevertheless, lower or even higher adiponectin levels were observed in cases compared to controls [6, 10]. These conflicting results may be explained by various genetic or environmental factors of the participants or by the fact that studies have applied different methods such as selecting diverse inclusion and exclusion criteria or sample sizes. Adiponectin is an insulin-sensitizing protein; hypoadiponectinemia is associated with insulin resistance [7]. Insulin resistance has recently been suggested as a contributing factor in the development of acne [16]. Moreover, androgens can reduce adiponectin levels [7]. It seems that the most common reason for acne formation is the production of sebum in response to androgens [15]. Stimulating the production of anti-inflammatory cytokines has also been attributed to this adipocytokine [6]. Concerning the oxidant and antioxidant status, the current study indicated that acne patients had markedly higher MDA levels than controls, whereas no significant differences were found in TAC concentrations. Increased MDA levels were also a predictor of developing the disease. These results are similar to the findings by an earlier study that had investigated the presence of oxidative and nitrosative stress in acne patients. The results had shown higher MDA, nitric oxide and carbonyl content levels and lower superoxide dismutase and glutathione concentrations in acne patients than in controls [12]. Moreover, another investigation had revealed higher serum MDA levels and lower TAC and zinc concentrations in patients with facial acne compared with healthy participants [13]. It has been found that sebum composition changes in acne. For instance, in comedones, the amount of linoleic acid is substantially decreased. This fatty acid can inhibit the action of several reactive oxygen species (ROS) produced by neutrophils such as superoxide radical anion, hydrogen peroxide and hydroxyl radical. As a result, ROS rises in comedo lesions [25]. The present study revealed significantly lower HDL-C levels in acne patients in comparison with controls, while other lipid profile components were similar between the groups. In a recent study, the lipid profile was assessed in three sequential menstrual cycles in the luteal phase of severe acne patients. The findings showed considerably lower HDL-C levels in patients than in controls, whereas total cholesterol and LDL-C levels were higher [15]. Besides, in the study carried out by Balta *et al*. [16], total cholesterol and LDL-C levels were remarkably higher in patients with post-adolescent acne than in controls, although no differences were detected in HDL-C or triglyceride concentrations. It is discovered that the RETN gene polymorphisms that influence the level of resistin expression are closely associated with acne vulgaris, and those individuals with variant RETN genotypes have lower HDL-C levels. Alterations in the lipid profile can affect sebum composition that increases inflammation during acne development [26]. To our knowledge, the present research was the first organized study that directly expressed that acne patients may have significantly lower fiber intakes compared with controls; and higher fiber intakes could be a protective factor for the disease. In a previous study carried out by Smith *et al*. [27], a low-glycemic load diet improved acne in young men. One of the characteristics of the diet was the higher fiber content in the low-glycemic load group than in the control one. There is a possibility that the fiber alone could ameliorate the disease. However, the authors did not discuss this issue. Furthermore, the effect of a low-fat, high-fiber diet, along with regular aerobic exercise was investigated in postmenopausal women. The results demonstrated that insulin levels decreased and sex hormone-binding globulin levels increased after three weeks of intervention [28]. It has been reported that hyperinsulinemia can lead to increased androgens and insulin-like growth factor 1. These are the conditions which can be associated with acne [1]. These findings may provide a basis for recommending foods with high fiber contents such as fruits, vegetables, legumes and whole grains [21] to patients suffering from acne. Besides, this study showed no significant differences in energy and other nutrient intakes between the groups. Investigations that have explored individual nutrient intakes in acne patients are very scarce. In one of the studies that used three-day food records, females in the acne group had significantly higher energy intakes compared to controls, while there were no differences in macronutrients, fiber, vitamin A, vitamin E, zinc and selenium [18]. In another research that applied a 24-hour dietary recall, the excess of energy and carbohydrate intakes and low vitamin A, carotene and zinc intakes resulted in progression of acne severity in both genders [19]. Dietary approaches used to be considered worthless in dermatological treatment. Nevertheless, based on recent studies, it has been indicated that dietary modifications may affect the course of some skin disorders such as acne, and may be recommended as a treatment procedure. Additionally, systemic medications for dermatological disorders have side effects, and dietary alterations may lower such risks. Thus, dermatologists should be able to properly consult the patients [17]. This research also confirmed some earlier reports [18, 29] that had concluded that acne patients were more likely to have a family history of acne compared with controls. This is in line with the findings of a large twin study that had indicated acne is highly hereditary [30]. Observing no significant differences in weights or BMIs between the groups was also in accordance with previous investigations [5, 16, 18]. This study aimed to investigate merely females suffering from acne. Some differences exist between males and females in terms of pathophysiology and management of acne as well as the quality of life. For instance, acne in men is more dependent on sebum production; and systemic anti-androgens can only be prescribed in women [31]. Moreover, women with acne have a higher risk of major depression than men [32], which justifies the need for further investigations in this gender. The major strength of the present study was applying logistic regression models to eliminate potential confounders. On the other hand, one of the limitations was the considerably fewer number of patients with severe acne than those with the moderate form. Due to this inequality of sample sizes, no comparison was made between different severities of the disease. Furthermore, measuring all the serum nutrient levels along with their dietary intakes could provide this research with more comprehensive results.

## Conclusion

In conclusion, female patients with acne vulgaris are more likely to have oxidative stress, dyslipidemia and a family history of acne than do healthy individuals. These abnormalities may be considered in the disease pathogenesis and may provide a basis for the progression of treatment strategies. Moreover, lower dietary fiber intakes in these patients support the significant link between the diet and acne and suggest the utility of dietary modifications. Further studies, particularly randomized controlled trials or cohort studies, are required to confirm the results.

## Acknowledgment

The authors would like to thank Shiraz University of Medical Sciences for approving and funding the research (project number: 94-01-87-10411).

## Conflict of Interest

The authors declare no conflicts of interest.

**Table 1:** Sociodemographic, Acne History and Anthropometric Data of Females with Acne and Controls. Data Are Presented as Mean ± SD or Number and Percentage.

**Characteristics**	**Acne patients** **(n=40)**	**Controls** **(n=40)**	**P-value**†
**Age (y)**	22.48 ± 4.15	23.38 ± 4.16	0.22
**Marital status, n (%)**			0.14
Single/divorced	31 (77.5)	25 (62.5)	
Married	9 (22.5)	15 (37.5)	
**Educational degree, n (%)**			0.29
High school diploma or lower	13 (32.5)	7 (17.5)	
Associate’s or bachelor’s degree	18 (45.0)	23 (57.5)	
Master’s degree or higher	9 (22.5)	10 (25.0)	
**Monthly income level, n (%)**			0.25
<10,000,000 Rials	17 (42.5)	10 (25.0)	
10,000,000-20,000,000 Rials	12 (30.0)	15 (37.5)	
>20,000,000 Rials	11 (27.5)	15 (37.5)	
**Family history of acne, n (%)**			0.006^*^
Absent	18 (45.0)	30 (75.0)	
Present	22 (55.0)	10 (25.0)	
**Acne duration (y)**	6.45 ± 5.94	-	-
**Weight (Kg)**	54.41 ± 8.00	56.65 ± 7.71	0.21
**Height (m)**	1.61 ± 0.06	1.61 ± 0.05	0.68
**BMI (Kg/m** ^2^ **)**	20.96 ± 2.94	21.91 ± 2.37	0.12

† Independent-samples t-test or Mann-Whitney U-test for comparing the continuous variables and Chi-square test for comparing the qualitative data; * P <0.05. **BMI:** Body mass index

**Table 2:** Comparison of Biochemical Parameters in Females with Acne and Controls. Data Are Presented as Mean ± SD or Number and Percentage.

**Biomarkers**	**Acne patients** **(n=40)**	**Controls** **(n=40)**	**P-value**†
**25-hydroxyvitamin D (ng/ml)**	19.18 ± 11.03	25.75 ± 19.96	0.22
**Vitamin D deficiency, n (%)**	26 (65.0)	24 (60.0)	0.64
**Adiponectin (µg/ml)**	8.42 ± 2.08	8.22 ± 1.79	0.64
**MDA (µmol/L)**	5.39 ± 0.79	4.90 ± 0.86	0.01^*^
**TAC (mmol/L)**	1.11 ± 0.27	1.15 ± 0.21	0.47
**Triglycerides (mg/dl)**	113.03 ± 43.91	102.60 ± 35.30	0.25
**Total cholesterol (mg/dl)**	150.28 ± 22.89	154.30 ± 25.96	0.46
**LDL-C (mg/dl)**	87.55 ± 18.84	87.00 ± 21.50	0.90
**HDL-C (mg/dl)**	45.83 ± 6.46	50.58 ± 10.34	0.02^*^

† Independent-samples t-test or Mann-Whitney U-test for comparing the continuous variables and Chi-square test for comparing the qualitative data; * P <0.05. **MDA:** Malondialdehyde; **TAC:** Total antioxidant capacity; **LDL-C:** Low-density lipoprotein cholesterol; **HDL-C:** High-density lipoprotein cholesterol.

**Table 3:** Comparison of Daily Nutrient Intakes in Females with Acne and Controls. Data Are Presented as Mean ± SD.

**Nutrients**	**Acne patients** **(n=40)**	**Controls** **(n=40)**	**P-value**†
**Energy (Kcal)**	1756.52 ± 413.22	1866.04 ± 355.90	0.21
**Carbohydrate (g)**	255.88 ± 68.77	270.84 ± 57.76	0.30
**Protein (g)**	59.33 ± 13.69	64.02 ± 14.32	0.14
**Fat (g)**	55.08 ± 13.67	58.51 ± 15.88	0.30
**Fiber (g)**	14.78 ± 5.22	17.93 ± 4.85	0.007^*^
**Vitamin A (µg RE)**	590.95 ± 513.42	856.04 ± 675.63	0.10
**Beta-carotene (µg)**	299.13 ± 447.21	540.83 ± 656.96	0.29
**Vitamin E (mg)**	4.86 ± 2.18	5.40 ± 1.92	0.24
**Vitamin C (mg)**	89.73 ± 43.87	98.09 ± 46.21	0.41
**Selenium (µg)**	73.52 ± 19.58	78.00 ± 25.39	0.38
**Zinc (mg)**	7.66 ± 1.97	8.48 ± 2.01	0.07
**Vitamin D (µg)**	0.72 ± 0.66	0.85 ± 0.95	0.46

† Independent-samples t-test or Mann-Whitney U-test for comparing the groups; * P <0.05. **RE:** Retinol equivalent

**Table 4:** Multivariate Logistic Model to Predict Acne Vulgaris

**Variables**	**Odds ratio**	**95% CI**		**P-value**
		**Lower**	**Upper**	
**Marital status (Ref: Single/divorced)**	0.36	0.09	1.41	0.14
**Monthly income level (Ref: Lowest income)**				
Intermediate income	0.53	0.14	2.10	0.37
Highest income	0.46	0.09	2.29	0.34
**Family history of acne (Ref: Absence of family history)**	4.00	1.18	13.53	0.03^*^
**BMI**	0.86	0.69	1.07	0.17
**25-hydroxyvitamin D**	0.97	0.93	1.02	0.21
**MDA**	2.15	1.01	4.55	0.047^*^
**HDL-C**	0.94	0.87	1.01	0.11
**Protein**	0.99	0.94	1.04	0.58
**Fiber**	0.87	0.77	0.99	0.04^*^
**Vitamin A**	0.999	0.998	1.00	0.50

* P <0.05. **CI:** Confidence interval;** Ref:** Reference category; **BMI:** Body mass index; **MDA:** Malondialdehyde; **HDL-C:** High-density lipoprotein cholesterol.

## References

[1] Williams HC, Dellavalle RP, Garner S (2012). Acne vulgaris. Lancet.

[2] Knutsen-Larson S, Dawson AL, Dunnick CA, Dellavalle RP (2012). Acne vulgaris: pathogenesis, treatment, and needs assessment. DermatolClin.

[3] Gieler U, Gieler T, Kupfer JP (2015). Acne and quality of life - impact and management. J EurAcadDermatolVenereol.

[4] Schwalfenberg GK (2011). A review of the critical role of vitamin D in the functioning of the immune system and the clinical implications of vitamin D deficiency. MolNutr Food Res.

[5] Lim SK, Ha JM, Lee YH, Lee Y, Seo YJ, Kim CD (2016). Comparison of vitamin D levels in patients with and without acne: a case-control study combined with a randomized controlled trial. PLoS One.

[6] Cerman AA, Aktas E, Altunay IK, Arici JE, Tulunay A, Ozturk FY (2016). Dietary glycemic factors, insulin resistance, and adiponectin levels in acne vulgaris. J Am AcadDermatol.

[7] Nishizawa H, Shimomura I, Kishida K, Maeda N, Kuriyama H, Nagaretani H (2002). Androgens decrease plasma adiponectin, an insulin-sensitizing adipocyte-derived protein. Diabetes.

[8] Toossi P, Azizian Z, Yavari H, Fakhim TH, Amini SH, Enamzade R (2015). Serum 25-hydroxy vitamin D levels in patients with acne vulgaris and its association with disease severity. Clin Cases Miner Bone Metab.

[9] Ozuguz P, Kacar SD, Asik G, Ozuguz U, Karatas S (2017). Evaluation of leptin, adiponectin, and ghrelin levels in patients with acne vulgaris. Hum ExpToxicol.

[10] Karadag AS, Ertugrul DT, Takci Z, Bilgili SG, Namuslu M, Ata N (2015). The effect of isotretinoin on retinol-binding protein 4, leptin, adiponectin and insulin resistance in acne vulgaris patients. Dermatology.

[11] Bowe WP, Logan AC (2010). Clinical implications of lipid peroxidation in acne vulgaris: old wine in new bottles. Lipids Health Dis.

[12] Al-Shobaili HA, Alzolibani AA, Al Robaee AA, Meki AR, Rasheed Z (2013). Biochemical markers of oxidative and nitrosative stress in acne vulgaris: correlation with disease activity. J Clin Lab Anal.

[13] Awad SM, Morsy H, Sayed AA, Mohamed NA, Ezzat GM, Noaman MM (2018). Oxidative stress and psychiatric morbidity in patients with facial acne. J CosmetDermatol.

[14] Al-Shobaili HA (2014). Oxidants and anti-oxidants status in acne vulgaris patients with varying severity. Ann Clin Lab Sci.

[15] Arora MK, Seth S, Dayal S (2010). The relationship of lipid profile and menstrual cycle with acne vulgaris. ClinBiochem.

[16] Balta I, Ekiz O, Ozuguz P, Ustun I, Karaca S, DogrukKacar S (2015). Insulin resistance in patients with post-adolescent acne. Int J Dermatol.

[17] Katta R, Desai SP (2014). Diet and dermatology: the role of dietary intervention in skin disease. J ClinAesthetDermatol.

[18] Ismail NH, Manaf ZA, Azizan NZ (2012). High glycemic load diet, milk and ice cream consumption are related to acne vulgaris in Malaysian young adults: a case control study. BMC Dermatol.

[19] SiniavskiiIu A, Tsoi NO (2014). Influence of nutritional patterns on the severity of acne in young adults. VoprPitan.

[20] Ramli R, Malik AS, Hani AF, Jamil A (2012). Acne analysis, grading and computational assessment methods: an overview. Skin Res Technol.

[21] Mahan LK, Raymond JL: Krause's food & the nutrition care process. 14th ed. St. Louis, MO: Elsevier Health Sciences; 2016.

[22] Blouin V, Bouchard I, Galibois I (2011). Body mass index and food and nutrient intake of children with type 1 diabetes and a carbohydrate counting meal plan. Can J Diabetes.

[23] Kramer C, Seltmann H, Seifert M, Tilgen W, Zouboulis CC, Reichrath J (2009). Characterization of the vitamin D endocrine system in human sebocytes in vitro. J Steroid BiochemMol Biol.

[24] Faghih S, Abdolahzadeh M, Mohammadi M, Hasanzadeh J (2014). Prevalence of vitamin D deficiency and its related factors among university students in Shiraz, Iran.

[25] Briganti S, Picardo M (2003). Antioxidant activity, lipid peroxidation and skin diseases What's new. J EurAcadDermatolVenereol.

[26] Younis S, Blumenberg M, Javed Q (2016). Resistin gene polymorphisms are associated with acne and serum lipid levels, providing a potential nexus between lipid metabolism and inflammation. Arch Dermatol Res.

[27] Smith RN, Mann NJ, Braue A, Makelainen H, Varigos GA (2007). A low-glycemic-load diet improves symptoms in acne vulgaris patients: a randomized controlled trial. Am J ClinNutr.

[28] Tymchuk CN, Tessler SB, Barnard RJ (2000). Changes in sex hormone-binding globulin, insulin, and serum lipids in postmenopausal women on a low-fat, high-fiber diet combined with exercise. Nutr Cancer.

[29] Ghodsi SZ, Orawa H, Zouboulis CC (2009). Prevalence, severity, and severity risk factors of acne in high school pupils: a community-based study. J Invest Dermatol.

[30] Bataille V, Snieder H, MacGregor AJ, Sasieni P, Spector TD (2002). The influence of genetics and environmental factors in the pathogenesis of acne: a twin study of acne in women. J Invest Dermatol.

[31] MoradiTuchayi S, Makrantonaki E, Ganceviciene R, Dessinioti C, Feldman SR, Zouboulis CC (2015). Acne vulgaris. Nat Rev Dis Primers.

[32] Yang YC, Tu HP, Hong CH (2014). Female gender and acne disease are jointly and independently associated with the risk of major depression and suicide: a national population-based study. Biomed Res Int.

